# Wild Ungulates as Disseminators of Shiga Toxin-Producing *Escherichia coli* in Urban Areas

**DOI:** 10.1371/journal.pone.0081512

**Published:** 2013-12-11

**Authors:** Alan B. Franklin, Kurt C. VerCauteren, Hugh Maguire, Mary K. Cichon, Justin W. Fischer, Michael J. Lavelle, Amber Powell, J. Jeffrey Root, Elaine Scallan

**Affiliations:** 1 United States Department of Agriculture, National Wildlife Research Center, Fort Collins, Colorado, United States of America; 2 Laboratory Services Division, Colorado Department of Public Health and Environment, Denver, Colorado, United States of America; 3 Division of Biostatistics and Bioinformatics, National Jewish Health, Denver, Colorado, United States of America; 4 Department of Epidemiology, Colorado School of Public Health, Aurora, Colorado, United States of America; Charité, Campus Benjamin Franklin, Germany

## Abstract

**Background:**

In 2008, children playing on a soccer field in Colorado were sickened with a strain of Shiga toxin-producing *Escherichia coli* (STEC) O157:H7, which was ultimately linked to feces from wild Rocky Mountain elk. We addressed whether wild cervids were a potential source of STEC infections in humans and whether STEC was ubiquitous throughout wild cervid populations in Colorado.

**Methodology/Principal Findings:**

We collected 483 fecal samples from Rocky Mountain elk and mule deer in urban and non-urban areas. Samples testing positive for STEC were higher in urban (11.0%) than non-urban (1.6%) areas. Elk fecal samples in urban areas had a much higher probability of containing STEC, which increased in both urban and non-urban areas as maximum daily temperature increased. Of the STEC-positive samples, 25% contained *stx*1 strains, 34.3% contained *stx*2, and 13% contained both *stx*1 and *stx*2. Additionally, *eaeA* genes were detected in 54.1% of the positive samples. Serotypes O103, and O146 were found in elk and deer feces, which also have the potential to cause human illness.

**Conclusions/Significance:**

The high incidence of *stx*2 strains combined with *eaeA* and *E-hyl* genes that we found in wild cervid feces is associated with severe human disease, such as hemolytic uremic syndrome. This is of concern because there is a very close physical interface between elk and humans in urban areas that we sampled. In addition, we found a strong relationship between ambient temperature and incidence of STEC in elk feces, suggesting a higher incidence of STEC in elk feces in public areas on warmer days, which in turn may increase the likelihood that people will come in contact with infected feces. These concerns also have implications to other urban areas where high densities of coexisting wild cervids and humans interact on a regular basis.

## Introduction

In 2008, eight children playing on a soccer field in Evergreen, Colorado were sickened with the same strain of Shiga toxin-producing *Escherichia coli* (STEC) O157:H7; five of these children were subsequently hospitalized. Ultimately, the source of these infections was genetically linked to feces from wild Rocky Mountain elk (*Cervus elaphus*), which used the soccer field for foraging [1]. In 2011, 5% of the elk feces collected from the same field and a nearby golf course were positive for non-O157 STEC, a level 2–3 times higher than previously published estimates for wild cervids [2], [3]. These two events motivated the questions: Are wild cervids a potential source of STEC infections in humans in Colorado and are STEC infections in cervids ubiquitous throughout the wild population?

While *E. coli* is common in human intestinal flora and is generally non-pathogenic, STEC are strains of *E. coli* that produce potent cytotoxins referred to as Shiga toxin (*stx*), which are further characterized as *stx*1 and *stx*2 [4]. *Stx*2-producing strains, especially when coupled with expression of *eaeA* and *E-hyl* genes, are considered to have a high likelihood of causing severe disease in humans [4]. STEC have been implicated in many high-profile outbreaks of disease in humans and cause an estimated 265,000 clinical cases of enteric illnesses, 3,700 hospitalizations and 31 deaths in humans in the U.S. each year though the origins of these infections are incompletely understood and rarely attributed to a specific source [5]. STECs are estimated to result in about $280 million in costs of illness each year [6]. Thus, determining the sources of STEC infections is critical to developing effective, evidence-based public health interventions. While the public health focus is often on O157 STEC because of its pathogenicity, non-O157 STEC serogroups caused twice as many acute infections in humans in the U.S. as O157 STEC [6]. Non-O157 serogroups primarily implicated in human disease include O26, O103, O111, O121 and O145 [4]. Both O157 and non-O157 STEC infections are a particularly important problem in Colorado where infection rates in humans are substantially higher than the national average [7]. Colorado also has the largest elk population in the U.S. [8] with elk and mule deer (*Odocileus hemionus*) living in proximity to humans in many urban and suburban areas. They also frequent agricultural areas, recreational parks, and green spaces. Although the 2008 outbreak documented an association of wild cervids with STEC transmission to humans in Colorado, the magnitude and risk has never been adequately demonstrated. Outbreaks of STEC in humans have also been linked to feces of wild cervids elsewhere, most recently in Oregon where deer feces may have contaminated strawberries with STEC [9].

While STEC has been identified in feces of deer and elk [3], [10]–[14], it is unknown whether their carriage of STEC is geographically uniform or is higher in animals living near humans or agriculture. For example, deer and elk could acquire STEC through contact with free-ranging cattle, drinking contaminated water, or foraging in urban and suburban green spaces irrigated with untreated water. If deer and elk acquire STEC from other sources, they may be intermediate reservoirs for STEC in a complex transmission cycle rather than the ultimate source for STEC infections in humans. To initially examine this issue, we addressed whether the prevalence and microbial serotypes of STEC in wild elk and mule deer in urban/suburban areas were similar to those in more remote areas of Colorado. Addressing this question begins to elucidate whether wild cervids are proximate or ultimate sources of STEC and identify whether potential spillback of STEC into elk populations occurs in areas of human habitation.

## Methods

### Ethics Statement

Permissions were obtained for all private areas that were sampled in this study.

### Sampling

From August through December 2012, we sampled wild cervid feces at 4 sites within areas used by wild elk that had a low likelihood of interchange with urban areas (Wild areas), 2 sites that were similar to wild areas but had evidence of use by free-ranging livestock (Livestock areas), and 6 sites within the towns of Estes Park and Evergreen (Urban areas). In the urban areas, the sites sampled included areas used by town residents, such as public parks and school grounds. Cervid feces were collected using disposable gloves and deposited in sterile Whirl-Paks®. Disposable gloves were changed before collecting each sample of ≥5 fecal pellets from each fecal pellet group to prevent cross contamination among fecal samples. A descriptive scale was used to age feces [15] and facilitate the collection of only fresh fecal samples. Samples were placed on ice in the field and sent on the day of collection to the Laboratory Services Division of the Colorado Department of Public Health and Environment (CDPHE), Denver, Colorado for analysis.

### Laboratory Analysis

Fecal samples were placed in 10 mL of GN enrichment broth (BD BBL™, Franklin Lakes, New Jersey) and incubated at 37°C in a rotating/shaking incubator for 18–24 hours. DNA was extracted from the enrichment broth culture using the MagNA Pure LC 2.0 (Roche Diagnostics, Indianapolis, Indiana). Using primers and the PCR protocol described by Reischl et al [16], nucleic acid extracts from the broth cultures were analyzed by PCR for the presence of the genes producing *stx*1 and *stx*2 Shiga toxin [16]. Broths yielding positive findings by PCR for Shiga toxin genes were cultured using MacConkey Agar with Sorbitol (Becton Dickinson, Franklin Lakes, New Jersey) and CHROMagar™ STEC (CHROMagar, Paris, France) agarose plates in order to isolate the toxigenic *E. coli* colonies. Multiple colonies were selected for repeat PCR testing to confirm the presence of *stx* genes prior to identifying O157 or non-O157 serogroups. Upon isolation of the Shiga-toxin producing colonies, PCR for *eaeA* and *E-hyl* genes was also performed on each isolate [16]. Shiga toxin-producing isolates were also characterized by group-specific latex agglutination. Pure bacterial cultures were grown 18–24 hours at 37°C in Brain Heart Infusion Broth (Becton Dickinson, Franklin Lakes, New Jersey). Broth cultures were boiled for 1 hour in order to remove capsular (K) antigens. Cultures were tested with *E. coli* monospecific antisera (Statens Serum Institut, Copenhagen, Denmark) according to package instructions using the microtiter plate method. Microtiter plates were incubated at 52°C overnight and reactions were read as positive or negative.

### Statistical Analysis

We analyzed the presence or absence of STEC in wild cervid feces using generalized linear models with a binomial distribution and logit link function using PROC GENMOD [17] in program SAS® (SAS Institute, Carey, North Carolina). We used a model selection framework to assess multiple competing statistical models using a version of Akaike’s Information Criterion adjusted for small sample sizes (AICc) [18]. The models we examined included effects of area type (urban vesus non-urban), month, season (summer [August], fall [September and October], and winter [November and December]), and maximum daily temperature (°C), which was the maximum temperature on the day of collection from the nearest NOAA National Climatic Data Center (http://gis.ncdc.noaa.gov/map/viewer/) weather station to the site of collection.

## Results

**Figure 1:**
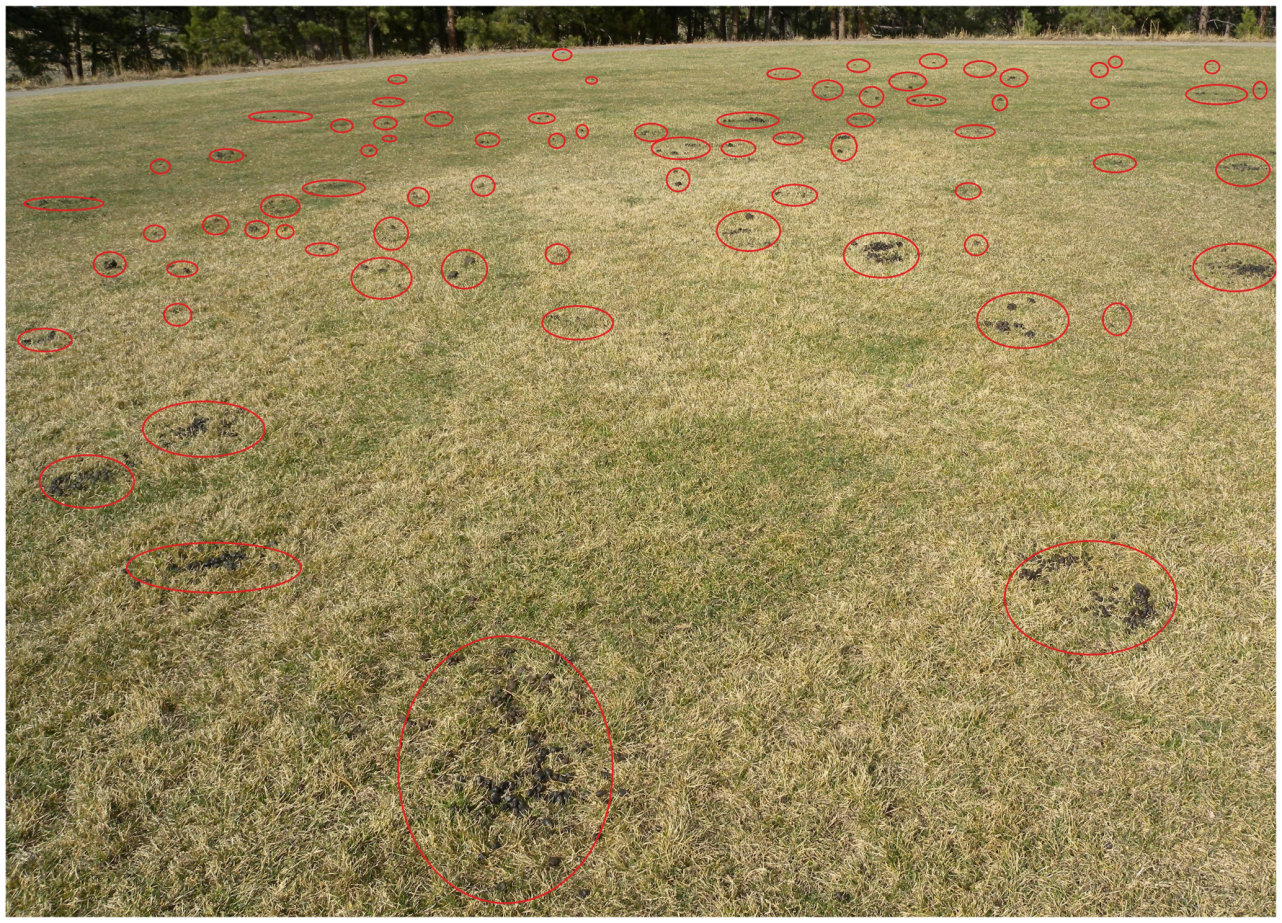
Example of high density of elk feces on an urban playing field in Colorado. Red circled areas denote locations of fresh elk feces deposited the previous evening.

We collected samples from 483 pellet groups from elk and mule deer in wild areas (12.0% of samples), wild areas with evidence of livestock grazing (26.1% of samples), and urban areas (61.2% of samples). The percentage of samples positive for either *stx*1 or *stx*2 (*n = *36) was much higher in urban areas (11.0%) than either wild areas (0%) or areas used by free-range cattle (2.4%) (Table 1). Fecal samples from mule deer were a small percentage (3.1%) of the total samples collected but appeared to have a higher incidence of positive samples in livestock and urban areas than elk (Table 1). However, the sample size from mule deer was too small to make sound inferences. In urban areas, elk commonly used public recreation areas where we sampled and deposited high densities of fecal pellet groups (Figure 1).

**Table 1:** Percentage of fecal samples from wild cervids positive for *stx* genes in three types of areas.

Species	*n*	Wild	Livestock	Urban	Overall
Mule deer	15	0.0 (–)	28.6 (0.0,62.0)^a^	50.0 (0.0,100.0)	20.0 (0.0, 40.2)
Rocky Mountainelk	468	0.0 (–)	0.8 (0.0,2.5)	10.8 (7.2,14.3)	7.1 (4.7, 9.4)
Overall	483	0.0 (–)	2.4 (0.0,5.0)	11.0 (7.5,14.6)	7.5 (5.1, 9.8)

a 95% confidence limits (dash indicates CL were not estimable).

**Figure 2:**
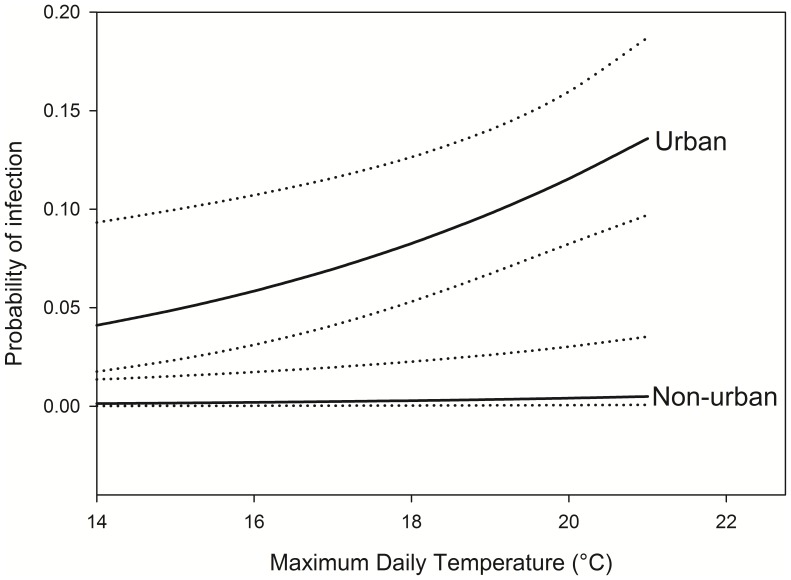
Effects of area type and maximum daily temperature (°C) on probability of infection of elk fecal pellets with Shiga toxin-producing *Esherichia coli*. Solid lines are estimates and dotted lines are 95% confidence limits.

Because of the small sample from mule deer, we modeled the probability of STEC infected fecal samples in elk only. Based on minimum AICc values, the model best explaining these data included the additive effect of whether samples were collected from urban or non-urban areas (samples from wild and livestock areas combined) and the maximum daily temperature on the day samples were collected (Table 2). This model was heavily weighted, based on Akaike weights. The top two models, which differed only by whether there was an interaction, accounted for almost all (98.1%) of the Akaike weights for the set of models examined. Under the top-ranked model, elk fecal samples in urban areas had a much higher probability of containing organisms with *stx* genes (*
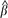
* = 3.46, 95% CL = 1.41, 5.51), which increased on both urban and non-urban areas as maximum daily temperature increased (*
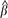
* = 0.19, 95% CL = 0.06, 0.31) (Figure 2). In terms of the two urban sites we sampled, *stx* genes were detected in 8.7% (95% CL = 2.0, 15.3%) of the samples from Estes Park and 13.7% (95% CL = 8.9, 18.5%) of the samples from Evergreen.

**Table 2:** Model selection results for examining the relationship between presence of *stx* genes in wild elk fecal samples and area type (urban vs non-urban), season, month, and maximum daily temperature (°C).

Model^a^	K	AICc	ΔAICc	Akaike Weight
Area type + Maximum dailytemperature	3	210.91	0.00	0.611
Area type × Maximum dailytemperature	4	211.92	1.01	0.369
Area type	2	219.32	8.40	0.009
Area type + Season	4	221.02	10.10	0.004
Area type + Month	3	221.28	10.37	0.003
Area type × Month	4	222.49	11.58	0.002
Area type × Season	6	224.34	13.42	0.001
No effects (intercept only)	1	240.65	29.74	0.000

a Model notation: + = additive main effects, × = main effects and their interactions.

Of the 36 fecal samples where *stx* genes were detected, three (8.3%) were from mule deer; two of these were *stx*1 (one from an area with free-range livestock and one from an urban area) and one was *stx*2 from an area with free-ranging livestock. Of the 33 fecal samples with positive findings for *stx* genes in elk, 32 were from urban areas and one was from a wild area used by free-ranging livestock (*stx*2). In the urban sites, all of the positive samples from Estes Park were *stx*2 positive while *stx*1 and *stx*2 genes were found in 80.8% and 69.2% of the positive elk fecal samples from Evergreen, respectively (Table 3). In addition, 50% of the positive elk fecal samples in Evergreen contained both *stx*1 and *stx*2 genes. Based on odds ratios, *stx*2 genes were 3.77 (95% CL = 1.12, 12.75) times more likely in elk feces in Estes Park than in Evergreen.

**Table 3:** Percentages of *stx* gene variants found in positive elk fecal samples from two urban sites in Colorado.

*Stx* Gene	Estes Park (*n* = 6)	Evergreen (n = 26)
*Stx*1	0.0 (–)	30.8 (0.0, 62.8)^a^
*Stx*2	100.0 (–)	19.2 (0.0, 53.8)
*Stx*1 & *stx*2	0.0 (–)	50.0 (22.8, 77.2)

a 95% confidence limits (dash indicates CL were not estimable).

Of the 36 samples positive for stx, we were able to culture isolates from 24 of those samples for the detection of *eaeA* and *E-hyl* genes. Of these, 66.7% of the *stx*1 PCR-positive samples, 30% of the *stx*2 PCR-positive samples, and 75.0% of the samples PCR-positive for both *stx*1 and *stx*2 contained *eaeA* genes, *E-hyl* genes or both (Table 4). All but one of the 24 samples contained the *eaeA* gene (Table 4). Of the 6 samples we were able to serologically identify, four were O146, one was O103.

**Table 4:** *EaeA* and *E-hyl* genes detected and serogroups for *stx* gene variants found in elk fecal samples from two urban sites in Colorado.

		Number of samples			
*Stx* Gene	*n*	*eaeA*	*E-hyl*	Both	Serogroups
*Stx1*	6	1	1	2	O103
*Stx2*	10	1	0	2	O146
*Stx1* & *stx2*	8	1	0	5	–

## Discussion

Both direct and indirect contact with animals has been implicated for causing both O157 and non-O157 STEC illnesses in humans [19], [20]. Indirect contact with wild cervids infected with STEC or ingestion of contaminated game meat have been implicated in at least 9 outbreaks of STEC in humans since 1995 (www.marlerblog.com/uploads/image/Deer%20Table%5B1%5D. pdf). In addition, a number of studies have found non-O157 STEC prevalence rates of 15.0−52.5% in individual wild cervids [11], [12], [21] and incidence rates of 19.4% in fecal samples from wild cervids [13]. Incidence rates of O157 STEC in fecal samples from wild cervids are much lower, ranging from 0.0−2.4% [3], [10], [14]. Our estimate of incidence rates of non-O157 STEC in wild elk, the focus serogroups in our study, was at the lower end of this range. However, our limited data on mule deer suggest higher incidence in this species, which warrant further examination. Additionally, not all these studies [11], [12] targeted the *eaeA* gene whereas in our study this gene was detected in 54.1% of the positive samples collected; this in combination with the presence of *stx*2-positive strains detected suggests the potential for transmission of these pathogens to humans. However, the potential for this to occur may be greater in specific geographic areas based on the genetic make-up of serogroups carried by a given elk herd. We previously documented an outbreak of human disease associated with direct contact with elk feces that occurred in one of the areas (Evergreen) we sampled in this study. Thus, prevalence or incidence rates alone of STEC in wild herds may not be a useful indicator for the potential to cause human disease; additional information on presence of *stx*1, *stx*2, *eaeA* and *E-hyl* genes is also required.

Our results strongly suggest that wild elk in urban areas have a higher incidence of STEC than those in either wild areas with little contact with urban areas or wild areas used by free-ranging livestock. There are a number of public health concerns resulting from our findings. First, we found a high incidence of *stx*2 combined with *eaeA* and *E-hyl* genes in wild cervid feces. In general, *stx*2 strains are 1,000 times more cytotoxic in humans than *stx*1 strains or strains carrying both *stx*1 and *stx*2; presence of *stx*2 is also the most important virulence factor associated with severe human disease, such as hemolytic uremic syndrome [22]–[24]. Bacterial *stx2* strains that also have the *eaeA* and *E-hyl* genes are strongly associated with the capacity of these strains to cause severe human disease [25]. Although we did not find STEC serotypes that are commonly implicated in human disease, such as O157, we did find several serotypes that are recognized as causing human illness. Specifically, serotypes O103 and O146 were found in elk feces in our study. These serotypes were collectively 17% of isolates found responsible for causing human illness from non-O157 STEC in the U.S. between 1983 and 2002, in some cases with severe symptoms in humans comparable to those caused by STEC O157 strains [26].

Second, there is a very close physical interface between elk and humans in the two urban areas that we sampled. Both urban areas experience high elk populations with wild elk frequently foraging and resting in public areas, such as playgrounds, golf courses, and public parks [27], [28]. For example, Estes Park has a resident elk population of about 2,500 animals [28]. In addition, these elk populations have grown substantially in the last 20−30 years so the coexistence with human residents has been relatively recent [27]. Thus, there is increased potential for human contact with elk feces in public areas within these two Colorado towns.

Third, we found a strong relationship between ambient temperature and incidence of STEC in elk feces. This relationship has also been observed in studies on livestock [4] and may be a function of shedding rates or proliferation of STEC populations in feces once they have been deposited into the environment with conducive temperatures. Regardless of the mechanism, a higher incidence of STEC in elk feces in public areas on warmer days (when people tend to utilize those areas more frequently) will further increase the likelihood that people will become infected with STEC.

The question remains as to whether wild cervids, especially elk, are proximate or ultimate sources of STEC contamination of the environment. Given the localized distribution of STEC infections of wild cervids around urban areas, we hypothesize that they are proximate sources with the ultimate source being localized factors, such as water contaminated with bacteria. Water contamination is plausible because some of the areas where we collected feces were irrigated with untreated water from natural sources, such as nearby rivers. Wildlife feces, including those from wild cervids, can be significant contributors to *E. coli* contamination of water [29] and at least one human STEC outbreak in Wyoming was suspected from elk and deer feces contaminating drinking water [30]. However, the reverse situation where cervids are infected from water sources has not, to our knowledge, been documented. In addition, both communities where we sampled also have very high visitation rates by tourists from around the world. For example, Estes Park receives over 2 million visitors a year with peak visitation rates in July and August [31]. The coupling of environmental contamination of public areas grazed by urban elk through water contaminated by sewage systems used by tourists magnifies the public health problem through possible introduction of novel strains of STEC, some of which may be of foreign origin, and which may be subsequently spread through wild cervids. We are currently examining this hypothesis in more detail.

We have identified a number of factors that may contribute to increased contact by humans with STEC shed in wild cervid feces, with at least one outbreak attributed to this type of contact. We suggest that outbreaks may not occur on a regular basis but rather may occur sporadically as a “perfect storm” of optimal conditions occurring at the same time and place. This situation also has implications to other urban areas where high densities of coexisting wild cervids and humans interact on a regular basis, a phenomenon that is becoming more commonplace [32], [33].
